# Housing Experience in Gated Communities in the Time of Pandemics: Lessons Learned from COVID-19

**DOI:** 10.3390/ijerph19041925

**Published:** 2022-02-09

**Authors:** Omar S. Asfour

**Affiliations:** 1 Architecture Department, King Fahd University of Petroleum & Minerals, P.O. Box 2483, Dhahran 31261, Saudi Arabia; omar.asfour@kfupm.edu.sa or o.asfour@hotmail.com; Tel.: +966-13-8603594; Fax: +966-13-8603210; 2 Interdisciplinary Research Center for Construction and Building Materials, King Fahd University of Petroleum & Minerals, P.O. Box 2483, Dhahran 31261, Saudi Arabia

**Keywords:** COVID-19, gated community, infection, post-pandemic, urbanism

## Abstract

Gated communities constitute an important component of the contemporary city in many countries, and the adequacy of such communities as a housing option has attracted the attention of researchers and policy makers from various backgrounds. However, it is unclear how gated communities will be perceived and reconsidered following the COVID-19 pandemic and whether this type of community will become more common. Thus, this study aims to investigate housing experience of gated community residents during the pandemic with reference to the urban context of Saudi Arabia. To this end, the residents of a selected gated community were surveyed using a structured questionnaire to identify the urban and architectural design factors that have affected their housing experience during the pandemic compared to that of the residents of non-gated communities. It was concluded that despite the criticism gated communities have received, they offered a safer and more controlled housing environment during the pandemic from the residents’ point of view, which may create additional housing demands for this type of residential community in the future. This requires further investigation for ascertaining how this may affect the housing market dynamics and strategies.

## 1. Introduction

The coronavirus disease 2019 (COVID-19) was identified in December 2019 and has since spread both globally and rapidly, formally being declared a pandemic in March 2020. The main attendant symptom is respiratory illness. While it is possible to slow down the infection rate by wearing masks, adopting social distancing and using open and well-ventilated spaces are required [1]. COVID-19 was not the first pandemic, and, as numerous scientists suggest, it will likely not be the last. For example, in the 20th century, three influenza pandemics were recorded, the most severe being the Spanish Flu pandemic of 1918–1919, which resulted in 20–50 million deaths [2]. While combatting pandemics is clearly critical, it is also important to learn from them and to document our experiences for gaining a better understanding of the required prevention and intervention measures. This is not only limited to the medical and public health disciplines but also includes other disciplines, such as urban planning and design. One main objective of urban planning and design is the creation of resilient cities that protect people’s health and empower community feeling, with these aspects becoming more vital in times of hardship, such as during pandemics. Here, it is crucial to adopt a proactive approach rather than a reactive one to develop long-term strategies that will ensure our urban environments are more resilient in the face of future crises.

Despite the fact that the COVID-19 pandemic is still prevailing at the time of this study, researchers have investigated the potential interaction between our urban environment and the pandemic in terms of the potential mutual effects. In the process, specific strategies to ensure our built environments are sufficiently safe and adaptable to face any future pandemics were discussed. Megahed and Ghoneim [3] presented various lessons learned from the COVID-19 pandemic that should be considered in relation to post-pandemic urbanism, with the authors arguing that our built environments will never be the same following the pandemic. Within this context, many unanswered questions require further multidisciplinary research for incorporating additional security layers in our urban environments to effectively combat any future pandemics. This includes how the COVID-19 pandemic may have influenced the urban pattern, home design, public green spaces, transportation and work culture [4]. Thus, the main research aim of this study was to investigate the impact of architectural and urban design on the experience of gated community residents during the COVID-19 pandemic compared to those of non-gated communities, with specific reference to the urban context of Saudi Arabia. The study also aimed at determining the factors that attract some residents to gated communities during pandemics and investigating future resident preferences in this regard. This study first provides a literature review focused on the theoretical background of both gated communities and the pandemic and later presents the data collection methods and analysis of the results.

## 2. Literature Review

### 2.1. Urban Environments and COVID-19

Several studies have discussed the urban and socio-spatial implications of COVID-19 [3,4,5], and several urban issues regarding these implications must be addressed. This includes urban dynamics, urban space concepts and urban life requirements as well as their psychological impacts. This also includes the new living and working styles that have emerged due to the pandemic and their respective impacts, such as school closures and working from home, on the development trajectories of our cities. The intensive utilization of information technology in our daily lives has caused a major shift from the physical to the virtual environments for offering safe interactions among people and accommodating the new living and working cultures. This also requires a high level of adaptability, where buildings could be reused for other purposes when required, such as temporary housing or medical centers.

The existing literature has also placed a specific focus on the role of housing design in pandemic mitigation. In fact, housing plays an integral role in response to pandemics in terms of mitigating the effects and helping people cope with the consequences [3]. Peters and Halleran [6] recommended that the post-pandemic design of apartment buildings must prioritize several aspects, including window designs aimed at providing pleasant views for stress recovery and acceptable lighting levels as well as having access to nature and the outdoor environment, which could be enhanced through specific balcony designs. Moreover, attention should be paid to the indoor air quality and the natural ventilation, especially in terms of living rooms, and the housing unit size should be reassessed to avoid crowding and to facilitate social distancing. For example, the residents of public housing faced several problems during the lockdown periods, including overcrowding, which accelerated the spread of the virus [7]. In fact, studies have suggested that the demand for densely populated housing types has already started to decline in certain housing markets as a result of the pandemic [4]. Thus, it is important to update the current design practices pertaining to apartment buildings to include a larger housing unit area per person as well as more private outdoor spaces that are integrated into the housing unit design. This is expected to make these units safer and more attractive in the post-pandemic era [4].

### 2.2. Gated Communities and COVID-19

**Figure 1:**
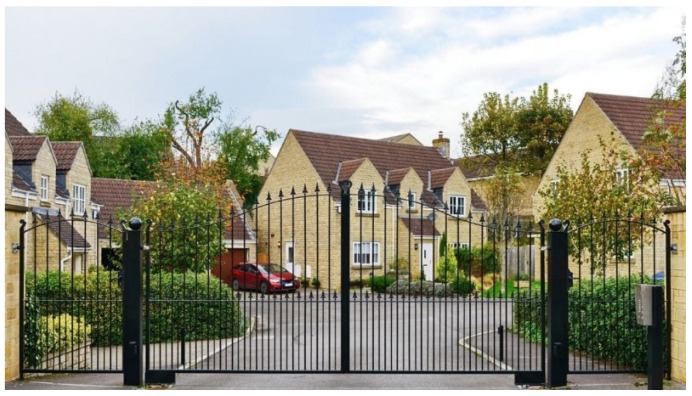
Gated communities form a significant component of the modern city [21].

In general, various types of communities can be identified as human settlements, which include rural, urban and suburban communities. Here, several sub-types are generally categorized under each type, including gated communities, which present a significant component of the modern city in many countries. Gated communities are also found in rural areas and are generally termed gated villages, while these have a different social context than their urban counterparts [8]. Furthermore, gated communities tend to have different physical characteristics, specifically in terms of urban closure aspects, such as the use of walls, restricted access using gates and the provision of a variety of collective services (Figure 1). Moreover, they have unique social characteristics, such as social homogeneity and a relatively weak relationship with the outside community [9] and have undergone rapid growth in recent decades due to their urban attractiveness. They include different typologies such as staff housing [10].

However, the spread of the pandemic started a debate surrounding the adequacy of gated communities. In fact, there is a body literature that discusses the relative advantages and disadvantages of gated communities. In general, people move to gated communities to gain certain advantages, such as greater privacy and security, attaining a specific lifestyle and satisfying the desire for prestige and reputation [11]. Furthermore, gated communities generally have less traffic due to the restricted access that prevents through traffic, which makes them both quieter and safer, especially for children. The advantages also include regular maintenance, community feeling and self-sufficiency, which have been considered as the basis of many urban models, such as the neighborhood unit [12]. This is enhanced through the social similarity among the residents and the availability of shared local services, which include recreational opportunities, attractive landscape features and open spaces. However, the debate surrounding these issues is still in progress. For example, it is suggested that the enhanced security is, in fact, overestimated with gated communities being potentially susceptible to new forms of risk depending on their urban design and socio-spatial configurations [13].

Meanwhile, the common disadvantages of gated communities include social segregation and the lack of contact with other social groups. The fact that these communities are inward oriented reduces their interaction with the rest of society, while certain studies have argued that the increasing number of gated communities in the modern city is directly related to the growth of the upper-middle class, which, in turn, exacerbates social inequality [14]. Of course, this is not essentially limited to the gated communities as urban inequality can also be found in open neighborhoods [15]. Other disadvantages include less mobility freedom, parking restrictions and higher housing unit prices compared to non-gated communities. The balance between these advantages and disadvantages requires taking a careful look at the urban and social context. For example, the disadvantages become more significant when a significant part of the population opts for moving to gated communities. In fact, a significant number of Saudi Arabian residents (estimated to be around a third of the population) live in gated communities [16], which include expatriate communities that are fairly common in Saudi Arabia, as they are believed to provide safer and more practical living environments. In addition, 80% of the residential communities in Shanghai are gated, a fact that led to certain policymakers calling for an end to gated communities by preventing the establishment of new ones and gradually opening up the existing ones [17,18].

However, this argument started to take another direction as a result of the current COVID-19 pandemic, with various studies suggesting that gated communities may become more popular after the pandemic, which is perhaps reflected by the increasing price of housing units within these communities. For example, the perceived advantage of greater security has already had a significant impact on the housing prices in China, reflected by an increase of 2% compared to the non-gated communities [19]. Residents believe that these communities are effective in pandemic risk reduction in terms of limiting outsider access, which creates a kind of ‘security zone’. However, there is a lack of direct medical evidence to conclude that gated communities are more effective in reducing the risk of COVID-19 infection [19]. In fact, Seanders and Maroofi [20] investigated the impact of gated communities on the spread of COVID-19 with reference to Jakarta, Indonesia, with the authors claiming that gated communities have the potential to slow down the spread of COVID-19 virus due to their relative self-sufficiency. However, further investigation and evidence is required in this regard. In general, the on-going COVID-19 pandemic has resulted in many significant shifts in our lifestyles, which has, in turn, influenced our housing preferences. These factors were further investigated through this research, as outlined in the following sections.

## 3. Materials and Methods

The main research aim of this study was to investigate the impact of architectural and urban design on the experiences of gated community residents during the COVID-19 pandemic in relation to those of non-gated community residents, including the factors that encourage or discourage people from living in a gated community. In this study, the gated community of King Fahd University of Petroleum and Minerals (KFUPM) in Dhahran, Saudi Arabia was selected as a case study, with a specific focus on the housing units. Based on the findings of this analysis, a survey of KFUPM faculty members was conducted using an online structured questionnaire. Because the students were evacuated from the campus at the beginning of the pandemic, they were excluded from the sampling, while the staff members were also excluded since many of them live off campus. A sample of non-gated community residents in the Eastern Province of Saudi Arabia was surveyed and compared with the results of the gated community survey.

KFUPM accommodates ~750 faculty members. The population of the Eastern Province is estimated to be ~5.2 million [22]. Assuming a confidence level of 95% and confidence interval of 10, the required sample size for both groups was 86 and 97 sampling units, respectively [23]. The questionnaire was then prepared, converted into a Google form and sent to both groups during the period of November 2021–January 2022 using different media until the required sample size was met. The responses were then analyzed using a statistical package for the social sciences platform and scored using a five-point Likert scale, with 5 indicating the highest agreement level. Meanwhile, a one-sample *t*-test was used to ascertain whether there was any significant difference between the means of one variable and the mid-value of the agreement five-point scale, that is, 3.0, while a two-sample *t*-test was also used to determine whether there was a significant difference between the means of two groups and the analysis of variance (ANOVA) technique was used to ascertain whether there was a significant difference between the means of more than two groups.

The questionnaire included the following four sections:

1. The first section was related to the respondents’ general information.
2. The second section included seven statements related to the impact of urban design on the residents’ experience of living in a gated or non-gated community during the pandemic:
  – Living in my gated community reduces the risk of infection as visitor access is controlled (this question was directed to the gated community residents only).
  – Living in my gated/non-gated community reduces the risk of infection because it is less crowded.
  – Living in my gated/non-gated community reduces the risk of infection because the residents strictly adhere to the COVID-19 preventative measures (masks, social distancing, etc.).
  – Living in my gated/non-gated community reduces the risk of infection because we are largely self-sufficient in terms of shopping facilities.
  – Living in my gated/non-gated community reduces the risk of infection because we are largely self-sufficient in terms of recreation facilities.
  – Living in my gated/non-gated community has provided more welfare and social support during the pandemic.
  – Living in my gated/non-gated community has not made me feel isolated during the pandemic.
3. The third section included five statements related to the impact of the housing unit design on the residents’ experience of living in a gated or non-gated community during the pandemic:
  – My house offers a sufficient area for online education (teaching/studying).
  – My house offers a sufficient area for social distancing if a family member gets infected.
  – My house offers healthy indoor spaces that are naturally ventilated.
  – My house offers healthy indoor spaces that provide good access to sunlight.
  – My house includes private outdoor spaces that proved highly useful during the lockdowns.
4. Finally, the fourth section included three statements related to the respondents’ post-COVID-19 housing preferences for gated or non-gated communities:
  – In general, during pandemics, I believe that it is better to live in gated communities than in open communities.
  – Based on my experience during the pandemic, I recommend that others move to a gated community.
  – If you were given the option, would you choose to live in a gated community in the post-COVID-19 era?

## 4. Results and Discussion

### 4.1. Housing Characteristics of the Case Study Subjects

**Figure 2:**
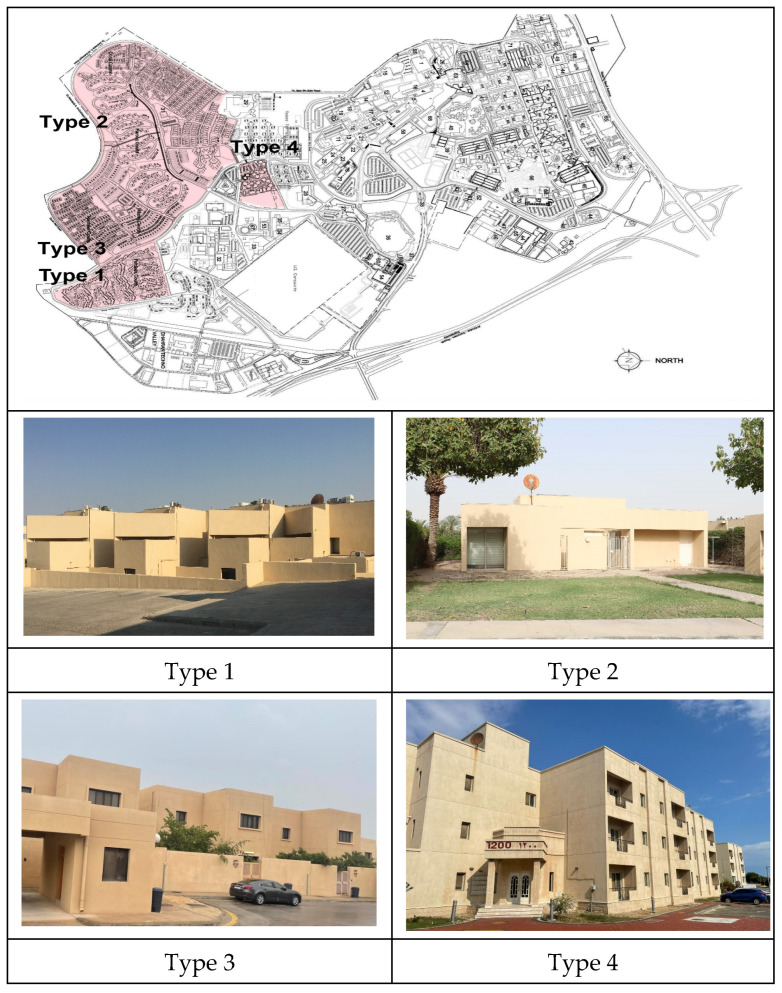
Top: King Fahd University of Petroleum and Minerals (KFUPM) site plan showing the faculty housing area in the highlighted zone [26]; bottom: different types of housing units at KFUPM.

While gated communities are common in many countries due to various socioeconomic factors, their emergence in Saudi Arabia was associated with the discovery of oil in the late 1930s. Here, well-maintained compounds were constructed to accommodate the expatriate professionals working in western companies while considering their cultural needs and backgrounds [24]. Following this, these communities become a common housing pattern available to all through the government and private sectors. As noted above, KFUPM in Dhahran was selected as the main case study subject. The adequacy of gated communities in any society is a context-oriented issue, one that is affected by various local factors, such as income level, housing prices in the market, security level and the culture and diversity of the society. KFUPM is one of the largest and well-established gated communities in the region and was established in 1963 [25]. Currently, the institution has a unique campus that includes academic and research buildings, faculty housing, student housing and various other facilities, including sports and shopping facilities (Figure 2); it also includes well-planned green areas and pedestrian walkways that are available to the residents during the pandemic as safe outdoor environments.

**Figure 3:**
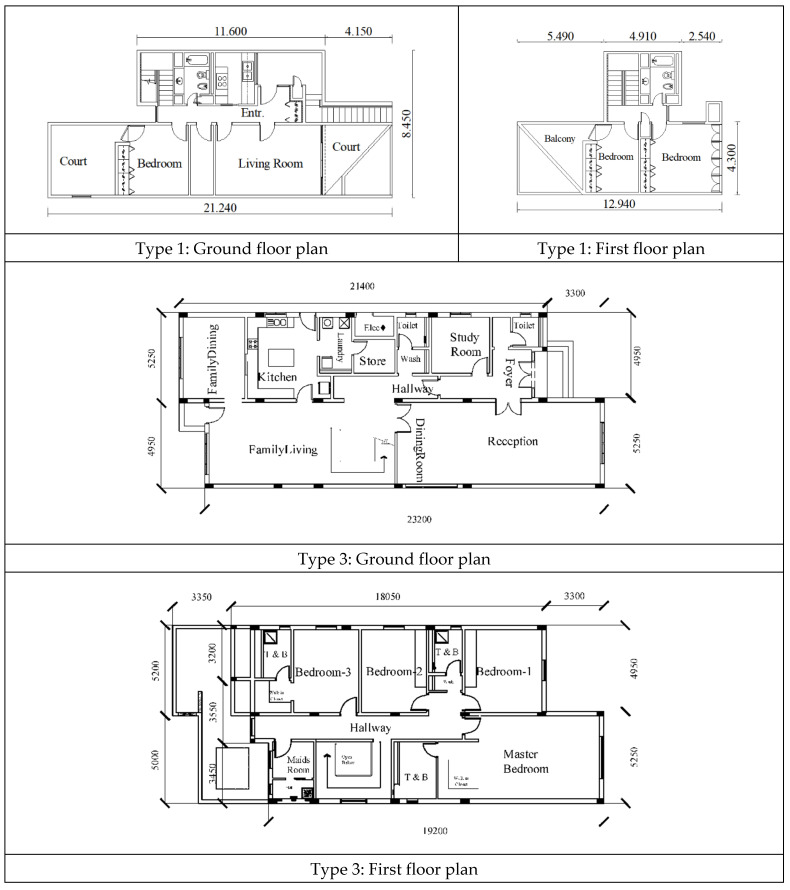
Typical floor plans of Type 1, and Type 3 [27] housing units in the surveyed gated community.

Furthermore, KFUPM campus includes several faculty residential zones that feature different urban design patterns, including the following four main types (Figure 2):

1. Type 1: Compact row houses arranged along several cul-de-sacs. Each group of units encloses a shared open space that includes vegetation and play areas. All units are bound together by their focus on this common open space, which creates a focused urban pattern.
2. Type 2: Single-floor detached houses arranged along several loop streets. Each unit includes its own private open space, which is maintained by the residents themselves. This urban setting creates a nodal urban pattern, which is used for separation.
3. Type 3: Two-floor detached villas arranged along various cul-de-sacs. Each unit includes its own private open space, which is used for separation. Figure 3 shows a comparison between the floor plans of this type and Type 1 (compact row houses). It shows a significant difference in housing unit area between these two types (about 418 m^2^ compared to 195 m^2^, respectively).
4. Type 4: Compact apartments for singles. Each group of units encloses a shared open space that includes vegetation; however, it does not include any private open space. Small balconies are integrated within the housing units.

### 4.2. Gated Community Survey Results

#### 4.2.1. Sample Characteristics

The results first revealed some general characteristics of the sample. Here, the respondents represented a variety of age groups as follows: 30–39 (31%), 40–49 (28%), 50–60 (20%) and over 60 (20%). They also live in different residential zones within KFUPM that include different housing types, with the most significant as follows: the compact row houses (the Al-Ferdaws courts) accommodating 34% of the respondents and the two-floor detached villas (the Al-Nakheel, Al-Murooj Al-Nafl and Al-Khuzama courts), accommodating 57% of the respondents. Finally, around one quarter of the respondents were local residents (Saudi nationals), while the remaining were international residents (non-Saudis).

#### 4.2.2. Impact of Campus Design

Section 2 of the questionnaire was aimed at investigating the impact of the campus design on the residents’ experience of living in gated communities during the pandemic. In general, the collected responses indicated that the respondents believe that living in their gated community reduces the risk of infection, which was attributed to the following:

1. Gated community has a controlled visitor access.
2. Gated community is less crowded.
3. The residents strictly adhere to the COVID-19 preventative measures (masks, social distancing, etc.).
4. Gated community is largely self-sufficient in terms of shopping facilities.
5. Gated community is largely self-sufficient in terms of recreation facilities.

Meanwhile, as shown in Table 1, the respondents believe that all these reasons are valid in terms of infection prevention because their mean agreement level exceeded 3. However, greater importance was given to the access control at the entry points of gated communities (relative importance index [RII] = 0.84, mean = 4.2), while the self-sufficiency of gated communities was regarded as the least important aspect (RII = 0.79 and 0.73 for the shopping and recreation facilities, respectively). This indicates that the residents of gated communities need to go outside their communities from time to time to satisfy their daily requirements and break their daily routine. Using a one-sample *t*-test, the mean of all the variables presented in Table 1 was observed to be 4.1 out of 5 (sig. = 0.0 < 0.05), which indicates that the respondents believe that these urban aspects reduce the risk of infection, thus making their gated community safer. An ANOVA test was adopted to ascertain the influence of age in this aspect, with the test indicating that there was no significant difference among the different age groups (sig. = 0.1 > 0.05).

Social cohesion and welfare are essential aspects for mitigating the fear related to pandemics, and the respondents presented their opinions regarding the impact of the campus design aspects on their social experience during the current outbreak. Here, less than half of the respondents (46%) believe that living in a gated community has provided greater welfare and social support during the pandemic, while 26% disagreed with this statement and the remainder (28%) were neutral. Meanwhile, the respondents were divided over the notion that they felt isolated in their gated community during the pandemic, with 38% agreeing with this statement and 38% disagreeing. Using the one-sample *t*-test, the mean of the social-experience-related questions was observed to be 3.1 out of 5; this indicates that the respondents are generally enjoying a fairly positive social experience, albeit that living in a gated community environment has made some feel isolated during the pandemic. This is consistent with the findings of AlQahtany [28], who surveyed a number of gated communities in Saudi Arabia and found that these communities generally enjoy good social relations, both internally and externally with the surrounding urban context. Further, the ANOVA test was adopted to ascertain whether age plays a role, with the test indicating that there was no significant difference among different age groups (sig. = 0.7 > 0.05). An independent sample *t*-test was used to ascertain whether nationality (local vs. international) has an impact, with the results indicating that the local residents have slightly less social satisfaction than the international residents (mean values = 2.98 and 3.15, respectively). However, this difference was statistically insignificant (sig. = 0.378 > 0.05).

#### 4.2.3. Impact of Housing Unit Design

Section 3 of the questionnaire was aimed at investigating the impact of the housing unit design on the residents’ experience of living in a gated community during the pandemic. KFUPM offers a variety of housing types based on specific basic needs, while there is also a pointing system in place that considers academic rank, work experience and other merits. This section included five questions related to specific essential housing design considerations that can determine the relative adequacy and safety in terms of health (Table 2). Using the one-sample *t*-test, the mean of all the variables presented was observed to be 3.6 out of 5 (sig. = 0.0 < 0.05), indicating that the respondents generally believe that their housing units fulfil the architectural design requirements in question, which proved to be useful during the pandemic. As shown in Table 2, the results indicated that 67% and 60% of the respondents believe that their houses have a sufficient area for online education and for social distancing, respectively. During the lockdown periods, many had to work and study from home using various online collaboration platforms. These platforms have become increasingly common even after the end of the lockdown, and it is expected that they will affect the post-pandemic working and learning patterns and, consequently, the attendant housing area patterns.

In terms of indoor environmental quality, 59% of the respondents believe that their houses offer healthy indoor spaces that are naturally ventilated and 67% reported that they have good access to sunlight. Finally, the majority of the respondents (70%) appreciate the fact that they have private outdoor spaces that were extremely useful during the lockdowns. Furthermore, the private open spaces could be effectively used for housing unit separation, which is consistent with the need for social distancing. However, the results indicated that a significant percentage of respondents (35%) are not happy with the floor area of their houses, considering the need for social distancing when a family member becomes infected. An ANOVA test was adopted to ascertain whether age had an influence on the response, with the results indicating that there was a significant difference among the different age groups (sig. = 0.0 < 0.05), with the difference being most notable between the younger age group (30–39) and the other groups. In fact, the younger age group reported being less satisfied with the design of their houses (mean = 2.6), which could be attributed to the fact that they have comparatively small families, making them eligible for smaller housing units. The ANOVA test was also used to ascertain whether the housing type has an impact on this aspect, with the results indicating that the residents of the Al-Ferdaws courts were less satisfied with the floor area of their housing units (mean = 2.2 out of 5). This was due to the compact nature of this housing zone, which was designed using comparatively small, attached units. However, the availability of a nearby central park proved to be highly useful for these residents during the pandemic who could go out walking while adhering to the social distancing measures. This confirms the importance of public outdoor spaces and walkways, which should be re-introduced in our future cities as an option during times of hardship.

#### 4.2.4. Post-COVID-19 Gated Communities

**Figure 4:**
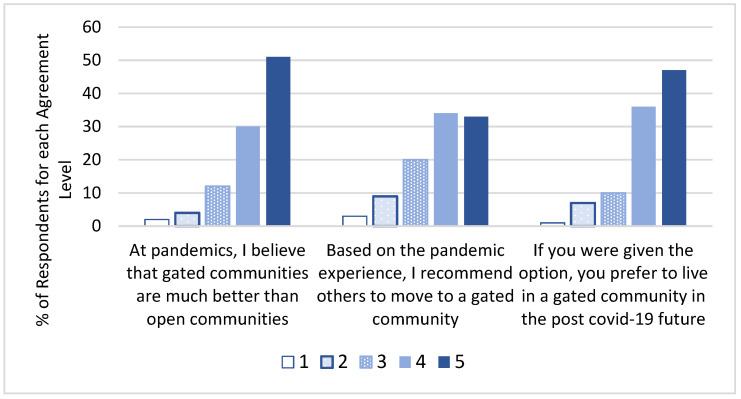
Gated community respondents’ feedback regarding post-COVID-19 gated communities.

Finally, the fourth section included three questions related to the respondents’ post-COVID-19 housing preference for living in a gated community (Figure 4). Here, the responses indicated that the majority of the respondents (81%) believe that it is better to live in a gated community than in an open community during pandemics, while a lower majority (68%) are willing to encourage others to move to a gated community. Finally, the vast majority (82%) also confirmed that if they were given the option, they would choose to live in a gated community, citing various justifications for this preference, including increased security, especially for children, increased safety during the pandemic due to the access control and less contact with crowds and the self-contained recreational facilities and comfortable living environment.

The above results indicate that the residents regard increased safety and security as major reasons for their preference for gated communities. This includes the external security (gates and walls), continuous documentation of visitors and internal security (security patrols). Over the last three decades, urban planners have largely focused on creating highly dense urban environments. However, this trend may become questionable in the post-COVID-19 era as more and more people seek less dense urban zones, such as gated communities. Meanwhile, a small number of respondents (8%) reported a preference for non-gated communities, citing various justifications for this preference, including the isolation from the nearby communities and the negative side effects at the societal level of living in a gated community, while they also questioned the assumption that access control is effectively preventing the spread of COVID-19.

### 4.3. Non-Gated Community Survey Results

**Figure 5:**
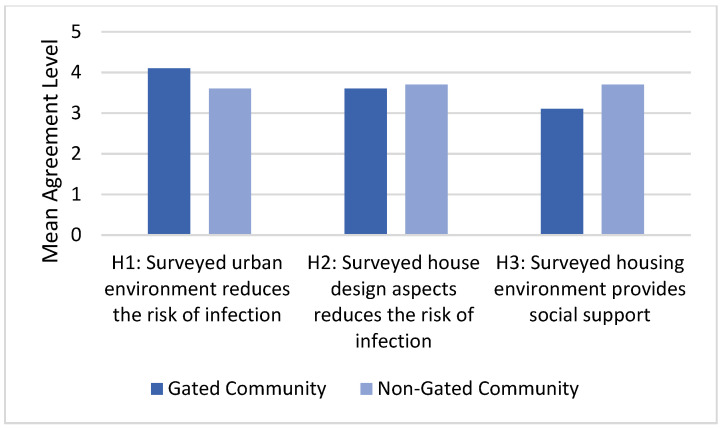
Comparison between the different study hypotheses in relation to gated and non-gated communities.

This survey was conducted considering a number of randomly chosen non-gated communities in the Eastern Province of Saudi Arabia with the aim of comparing the different study hypotheses in relation to gated and non-gated communities (Figure 5). In this survey, the respondents live in different locations within the Eastern Province of Saudi Arabia, with the majority living in Dammam (50%), Khobar (28%) and Dhahran (10%). Here, the residential areas include different housing types, including villas (32%) and flats (65%), while the age distribution was as follows: 20–29 (11%), 30–39 (46%), 40–49 (33%), 50–60 (9%) and over 60 (1%). Finally, around half of the respondents were local residents (Saudi nationals), while the other half were international residents (non-Saudis).

Similar to the gated community survey, the first section of this survey investigated the impact of the housing urban design on the residents’ experience of living in a non-gated community during the pandemic. Using a one-sample *t*-test, the mean of all the related aspects (low density, implementation of infection prevention measures and self-sufficiency) was observed to be 3.6 out of 5 (sig. = 0.0 < 0.05), which indicated that the respondents believe that these urban aspects reduce the risk of infection, thus increasing the safety of the community. However, the agreement level here was less than that among the gated community (4.1). As presented in Figure 5, this difference is more significantly attributed to the strict application of COVID-19 preventative measures (4.17 and 3.67 out of 5 for the gated and non-gated communities, respectively).

**Figure 6:**
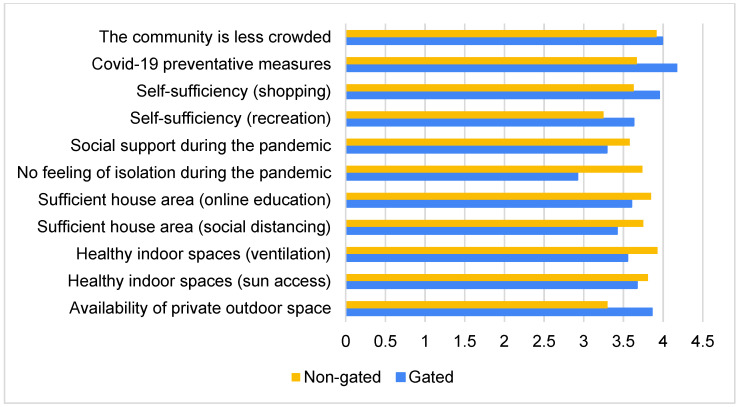
Respondents’ evaluation of the availability of some urban and architectural design aspects that affected their housing experience during the pandemic in both gated and non-gated communities.

Following this, the impact of the housing unit design on the residents’ experience of living in a non-gated community during the pandemic was investigated. Using a one-sample *t*-test, the mean of all the relevant housing design aspects (floor area, natural ventilation, access to sunlight and availability of outdoor spaces) was observed to be 3.7 out of 5 (sig. = 0.0 < 0.05). Much like with the gated community, this finding indicated that the respondents generally believe that their housing units fulfil the architectural design requirements in question, which proved to be useful during the pandemic. Furthermore, the agreement level (3.7) was almost the same as that obtained in the case of the gated community (3.6). However, Figure 6 shows that some differences could be observed between the gated and non-gated communities here. For example, the gated community provides more private space compared to the non-gated one (3.86 compared to 3.30 out of 5, respectively).

The survey also investigated the respondents’ social experience of living in a non-gated community during the pandemic, and, further using a one-sample *t*-test, the mean was observed to be 3.7 out of 5 (sig. = 0.0 < 0.05). Considering that the mean value was 3.1 in the case of the gated community residents, the non-gated community residents are clearly having a better social experience during the pandemic. As presented in Figure 6, this difference is mainly attributed to the feeling of isolation. Residents of the gated community showed significantly less agreement level to the notion that their community does not result in the feeling of isolation (2.92 and 3.74 out of 5 for the gated and non-gated communities, respectively). These findings also suggest that the surveyed non-gated community residents are generally having a fairly positive living experience in their communities during the pandemic. However, almost half (46%) believe that it is better to live in a gated community than in an open community during pandemics, reporting that, if they were given the option, they would choose to live in a gated community.

## 5. Conclusions

Despite the fact that the COVID-19 pandemic remains prevalent, a particular focus has been placed on the role of housing design and planning in mitigating its impact on people. Within this context, the adequacy of gated communities remains controversial. To many policymakers, in comparison to open communities, gated communities are perceived as a largely segregated urban form. However, it is suggested that gated communities may become more popular in the post-pandemic era due to their perceived role as a safe zone. Thus, this study was aimed at investigating the housing experience of gated community residents during the pandemic compared to that of non-gated communities. The study first discussed how our cities are responding to the challenges of COVID-19, how urban planning and design should address these challenges and the role of gated communities within this context. Following this, the residents of non-gated communities in the Eastern Province of Saudi Arabia and a specific gated community in the same region were selected as part of the case study (KFUPM campus) involving a structured questionnaire. The aim of the questionnaire was to identify the urban and architectural design factors that have affected the residents’ experiences during the pandemic.

In general, this study concluded that despite the criticism gated communities have received, they have provided a safer and more controlled housing environment during the pandemic from the residents’ point of view. In fact, the results revealed several reasons why residents may choose to live in gated communities during pandemics, with the RII analysis indicating that the most important factor was the controlled visitor access, followed by the strict adherence to COVID-19 preventative measures (masks, social distancing, sanitization, etc.) and the notion that gated communities are generally less crowded. Using various *t*-tests, it was found that the respondents from the gated community believe that these aspects reduce the risk of infection, thus making their community safer. In terms of the social aspects, it was found that living in a gated community has had a positive impact on the respondents’ social wellbeing during the pandemic; however, a feeling of isolation was a major concern.

To promote a healthy housing environment in gated communities, several recommendations related to housing design were suggested based on the lessons learned from the COVID-19 pandemic, including a sufficient area for online education, sufficient housing unit areas for social distancing if a family member becomes infected, good ventilation, access to sunlight and enhanced contact with the outdoor environment using private open outdoor spaces. The non-gated community survey indicated that the surveyed residents are generally enjoying a fairly positive living experience in their communities during the pandemic. However, around half of them believe that it is better to live in a gated community than an open community during pandemics, reporting that, if given the option, they would choose to live in a gated community.

Recommendations for further research are as follows:

1. The adequacy of gated communities in any society is a context-oriented issue and there currently exists a limited amount of literature based on this topic. Thus, it is recommended that additional studies are conducted in relation to other contexts to help expand the findings of this study.
2. To conduct additional investigations of the potential negative aspects of gated communities as individual and social protection solutions in the face of the pandemic.
3. To perform additional investigations using medical evidence to determine the impact of gated communities on the COVID-19 infection rate. It is also recommended that a review of housing design standards is presented, including the analysis of the minimum floor area required to satisfy the emerging online education and working requirements.
4. Finally, it is recommended that a market study related to the expected demand for gated communities in the post-pandemic era is conducted. This will be essential for determining how strategic housing planning and housing market dynamics will be affected.

## Figures and Tables

**Table 1:** Respondents’ rating regarding the importance of different infection prevention strategies in their gated community.

Agreement Level	Respondents’ Agreement Regarding the Infection Prevention Strategy (%)				
	Access to the Community Is Controlled	Gated Community Is Less Crowded	Preventative Measure Application	Self-Sufficiency (Shopping)	Self-Sufficiency (Recreation)
1	2	3	1	3	3
2	8	4	7	6	14
3	9	9	14	13	23
4	30	54	30	44	32
5	51	29	48	33	27
Total	100	100	100	100	100
Mean	4.2	3.99	4.17	3.95	3.63
RII	0.84	0.80	0.83	0.79	0.73
Rank	1	3	2	4	5

**Table 2:** Respondents’ evaluation of the availability of certain architectural design aspects in their gated community that have affected their housing experience during the pandemic.

No.	Housing Unit Characteristics	Respondents for Each Agreement Level (%)					Mean
		1	2	3	4	5	
1.	Sufficient area for online education (teaching/studying)	17	10	6	30	38	3.6
2.	Sufficient area for social distancing if a family member got infected	14	21	5	29	31	3.42
3.	Healthy indoor spaces that were naturally ventilated	10	14	17	29	30	3.55
4.	Healthy indoor spaces that have good access to sun	11	12	10	33	34	3.67
5.	Private outdoor spaces which were useful during lockdowns	9	4	17	32	38	3.86

## Data Availability

The data presented in this study are available on request from the corresponding author.
